# Literary Fiction Influences Attitudes Toward Animal Welfare

**DOI:** 10.1371/journal.pone.0168695

**Published:** 2016-12-22

**Authors:** Wojciech Małecki, Bogusław Pawłowski, Piotr Sorokowski

**Affiliations:** 1 Department of Literary Theory, Institute of Polish Philology, Faculty of Philology, University of Wrocław, Wrocław, Poland; 2 Department of Human Biology, Faculty of Biological Sciences, University of Wrocław, Wrocław, Poland; 3 Department of Experimental Social Psychology, Institute of Psychology, Faculty of Historical and Pedagogical Sciences, University of Wrocław, Wrocław, Poland; Eidgenossische Technische Hochschule Zurich, SWITZERLAND

## Abstract

Literary fiction has been credited with considerable power to improve attitudes toward outgroups. It was even argued that it has been an important factor behind the global decline of violence against various minorities in the last centuries. Could it also help to reduce the human-inflicted suffering of animals? To test this, we studied the attitude toward animal welfare of n = 921 (experimental group) people of both sexes who read a short fragment of an unpublished novel with a motif of the physical abuse of an animal. The control group (n = 912) read a fragment of a similar length but not related to animals. After reading the text all subjects filled out an on-line questionnaire with seven items (camouflaged among many others items) measuring attitudes toward animal welfare. The questionnaire included also demographical questions, such as whether the subject keeps pets. We found that in comparison with the control group, the experimental group was significantly more concerned about animal welfare. This result indicates that literary fiction can influence attitudes toward other species. It is also worth noting that our study is characterized by a high level of ecological validity, i.e. a relatively high extent to which its results can be generalized (or extended) to real-world settings. Due to its specific design, which involved the cooperation of a bestselling author and his publisher, the study approximated the typical conditions in which people read fiction in a remarkably accurate way. Finally, our research has potential practical implications for promoting animal welfare.

## Introduction

There is a growing body of evidence, from ethology, neuroscience, and other fields, indicating that non-human animals can experience suffering in ways similar to ourselves [1–3]. This research lends scientific support to calls from animal ethicists and activists alike to eliminate as unnecessarily cruel various widespread ways of treating other species, e.g., those involved in factory farming and laboratory research [4–6]. In practice, this goal cannot be achieved without first making the public more concerned about animal welfare [7]. It has been hypothesized that literary fiction might be of help here [8,9], and this option should be considered seriously. Literary fiction is a popular form of entertainment which can make almost any subject interesting to wide audiences. In contrast, pro-animal organizations often have to resort to scandalizing, or even to paying money to members of the public, in order to draw attention to material that they hope might raise concern for the welfare of countless farm and laboratory animals [10,11]. Moreover, fiction is also well-suited to providing vivid, emotionally-charged accounts of individual suffering. As argued by psychologists, such accounts are highly effective in raising public attention to large-scale misery [12,13], particularly in comparison to statistical or numerical data, on which animal welfare campaigns often rely. In fact, when the public is exposed to such data, this usually results in so-called psychophysical numbing, i.e. a collapse of compassion [12].

Speculation about the power of literary fiction to influence people’s concern about animals is generally encouraged by two kinds of data. First, there is anecdotal data on the social impact of famous animal-themed novels such as, e.g., Anna Sewell’s 1877 *Black Beauty*, a bestseller that is reported to have inspired a large number of people to join humane societies and show their support for anticruelty legislation, something which eventually led to outlawing certain kinds of practices harmful to horses [14]. Second, there is the historical and experimental research on the influence of fictional texts on our understanding of others and on their capacity to improve attitudes toward human outgroups such as homosexuals, immigrants, and refugees [15–22]. Still, no experimental research has yet been devoted to whether the impact of fiction extends to attitudes toward members of other species, and our study aimed to fill this gap in examining whether a fictional narrative can elicit greater concern for animal welfare.

## Materials and Methods

In order for our results to be practically relevant, we first had to ensure that our study had an appropriate level of ecological validity. For this purpose, we created a unique design that involved the cooperation of a large book publisher, a market research agency, and a bestselling author. The author in question was Marek Krajewski, one of the most popular Polish writers, who has also gained considerable recognition around the world, with his books having been translated into more than 20 languages.

### Procedure

Four weeks before the official publication date (Sept. 11, 2014) of Krajewski’s then latest novel, the author announced on his public Polish Facebook profile a quiz that served as a cover for our study (Announcement A and B in S1 Text). It was also announced on his publisher’s Facebook profile (Announcement C and D in S1 Text) and on the website of a popular Polish book lovers’ community. Within the period of the 19 days for which the experiment was running, the quiz attracted 1833 Polish readers, 89% of whom participated on the first three days.

The quiz offered an opportunity to read a fragment of Krajewski’s then still unpublished novel titled *Władca Liczb* (*The Lord of the Numbers*) and to win a free copy of the book. At our request the author included in the novel a narrative describing the exploitation and physical abuse of an animal (a monkey) by negatively depicted human individuals (S2 Text). We chose this topic as being potentially conducive to attitudinal change, based on existing research on the so-called empathy-altruism hypothesis and on the historical data concerning the impact of fiction on attitudes toward animals [14,23,24]. The participants were randomly assigned to each of the following two experimental conditions: the opportunity to read on-line a three-page (941-word) fragment of the novel that concerned the plight of the monkey (the experimental group) or an alternative fragment of similar length (947 words) in which the main protagonist of the novel is approached by a stranger with a request to solve an as yet unspecified case (S3 Text)–a subject we had deemed neutral from the perspective of our study (the control group).

Immediately after reading the text, the subjects filled out an on-line questionnaire whose ostensible purpose was to examine the psychological profile and worldview of Krajewski’s readers and their impressions about the text they read (S4 Text). The questionnaire consisted of 53 Likert-type items scored on a 7-point scale, where 1 meant ‘I completely disagree’ and 7 meant ‘I completely agree’. Camouflaged among items concerning personality traits as well as moral and political beliefs (e.g., “Cultural minorities should be protected and supported”), were seven items measuring attitudes toward animal welfare (e.g. “Human needs should always come before the needs of the animals”).

The scale was developed by the authors on the basis of the Animal Attitudes Scale (AAS) [25]. We decided to modify the original scale due to cultural specificity. The scale used in this study consisted of seven items, of which four were adopted from the AAS and three were added to directly measure attitudes toward animal welfare, e.g. “I feel personally responsible for helping animals in need” (S5 Text). Scores in the Attitudes Toward Animal Welfare (ATAW) scale, which is how we call our scale, could potentially range between 7 and 49 points– 7 questions marked on a 7-point Likert scale (Table 1). Analogically to the AAS, the higher score achieved on our scale is an indicator of pro-animal welfare attitude. Three items were reversed. A pilot study (N = 55) showed that the psychometric properties of the scale were appropriate. The reliability of the scale measured with Cronbach’s alpha was .81, while its validity (the correlation with AAS) was r = .7, p < .0001.

**Table 1:** Descriptive statistics for each of the ATAW scale items.

Attitudes Toward Animal Welfare (ATAW)	Min	Max	*M*	*SD*
1. The slaughter of whales and dolphins should be immediately stopped even if it means that some people will be put out of work	1	7	5.5	1.5
2. The suffering of animals is an acceptable price for inventing drugs for humans*	1	7	4.8	1.7
3. Human needs should always come before the needs of animals*	1	7	4.2	1.7
4. I feel personally responsible for helping animals in need.	1	7	4.6	1.5
5. The low costs of food production do not justify maintaining animals under poor conditions.	1	7	6.0	1.2
6. Apes should be granted rights similar to human rights	1	7	2.6	1.3
7. Basically, humans have the right to use animals as we fit*	1	7	5.9	1.2

Items marked with an asterisk (*) are reverse-scored.

At the end of the questionnaire participants provided demographical data, including whether the subjects keep pets.

A professional market research agency was hired to create and manage the on-line questionnaire, as well as to design a special website through which the questionnaire was accessed by the subjects. To minimize the risk of a given person participating in the study more than once, two measures were used: (a) http cookies that blocked access to the questionnaire from the same web browser once it had been filled out; (b) the verification of the personal data that the participants submitted in order to take part in the quiz.

In order to reduce the possibility of communication between the participants interfering with the results, they were asked to abstain, for the duration of the quiz, from revealing any details about the questionnaire or the texts they had read, including on the author’s Facebook profile. For the same reason, while the participants were given a chance to opt out of the study after completing the survey, they were not debriefed. To our best knowledge, no relevant details were revealed publicly and no participant expressed suspicion as to its real subject.

### Participants

The study involved 1833 participants: 1241 women, aged between 14 and 81 (M = 31.2 SD = 9.4), and 592 men, aged between 15 and 69 (M = 32.6, SD = 9.2).

### Ethics Statement

The experiment described in this paper was conducted according to the principles expressed in the Declaration of Helsinki and in full compliance with Facebook’s policy on collecting data from its users. It was approved by the Research Ethics Committee at the Institute of Psychology, University of Wroclaw. The Committee specifically waived the requirement for informed consent (due to the specific nature of the experiment, no informed consent could be obtained from the subjects), approved the inclusion of minors (<18 years old) in our study, and the lack of informed consent for these participants, and reviewed and approved the deceptive aspects of the study, including the lack of debriefing.

## Results

To verify whether our experimental setting influenced attitudes toward animal welfare, we performed analysis of variance (ANOVA) with pairwise comparisons. All analyses were obtained by the means of Statistica Software, version 12.

**Fig 1:**
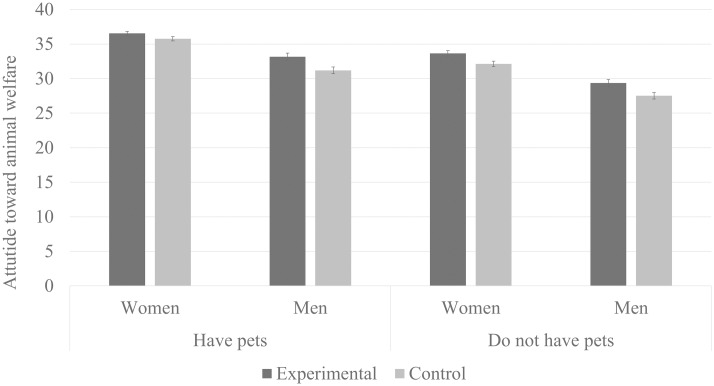
The influence of the experimental conditions on Attitudes Toward Animal Welfare (error bars represent standard error vales).

The mean score in ATAW scale for the whole sample was *M* = 33.4 (SD = 6.7), with minimal value of 7 and maximal of 49 points. We performed analysis of variance (ANOVA) with participants’ sex (male vs. female), pet possession (possessing vs. not possessing) and condition (experimental vs. control) included as independent variables and ATAW score as the dependent variable. Tested model revealed a significant main effect of participants’ sex, F_1,1825_ = 189.9, *P* < .0001, *η*^2^ = .09. Pairwise comparisons indicated that women (*M* = 34.5±.2) expressed more pro-animal welfare attitudes than men (*M* = 30.3±.25). There was also a significant main effect of pet possession, F_1,1825_ = 131.1, *P* < .0001, *η*^2^ = .07, indicating that participants who declared having a pet at home scored higher in ATAW (*M* = 34.2±.21) as compared to those who did not report possessing a pet (*M* = 30.7±.22). We also found a significant main effect of the condition, F_1,1825_ = 25.1, *P* < .00001, *η*^2^ = .02, indicating that the participants from the experimental group (who read the text about the abused monkey) scored higher in ATAW (*M* = 33.2±.22) than participants from the control group (*M* = 31.7±.21) (see Fig 1). No significant interaction effects were observed (all *F*s < 1.6 *p*s > .2).

In order to address the potential worry that the inclusion in our questionnaire of an item concerning apes (see Table 1) might have skewed the results of our study (which used a text about a monkey) we have performed additional analyses which excluded that particular item. The general results were the same as reported above.

## Discussion

The obtained data confirm that the fictional narrative used in our experiment influenced the subjects’ attitudes toward animal welfare in the sense of making their attitudes more pro-animal welfare. Since this effect was observed in almost all tested groups, even in the group that initially presented the least pro-animal welfare attitudes (i.e. men not having pets), our data also confirm that the effect was not due to a specific sample that is sensitive to the well-being of animals in general.

Moreover, the obtained data corroborate the results of experimental studies which show that fictional and non-fictional narratives depicting the plight of an individual member of a given group (e.g. a drug addict) can help improve attitudes toward that group as a whole [21,24]. In our case the effect is particularly striking given that “animals” are an unusually broad and diverse group. Our experimental narrative concerned the abuse suffered by a single monkey, and yet it had an effect on attitudes toward animals in general, measured with a scale that included items that were unrelated to the particular case from the narrative such as “The slaughter of whales and dolphins should be immediately stopped even if it means that some people will be put out of work” (Table 1).

Our results become even more significant when one considers how our experiment approached the question of ecological validity, which is often dealt with in a rather unsatisfying way by empirical studies on fiction reading. It has been pointed out [13,26], for instance, that such studies often rely on textoids instead of actual fictional texts or make subjects read fiction in unusual, laboratory conditions. The design we employed allowed us to avoid these and other problems. We used a genuine fictional text and performed our study outside of laboratory settings. It may be assumed, too, that our subjects were genuinely interested in reading the text and following its plot, and that they would have been inclined to read it independently of the experimental conditions. In addition, it is now an established practice that authors publish on-line sample material from their forthcoming books for marketing purposes, so the fact that the participants read only a fragment of the novel and that they did so from their computer or tablet screens most likely did not seem extraordinary to them.

Note also that the design of our study made it practically impossible for the subjects to guess its purpose, therefore minimizing the risk of the so-called subject-expectancy effect occurring. It has also allowed us to gather responses from more than 1800 individuals of diverse age and educational background, while research in the general field of attitudinal effects of literary fiction has thus far relied on samples numbering up to approximately one hundred people, most of them university students [27]. Furthermore, as Krajewski specializes in a particularly popular fiction genre, i.e. mystery/detective [28], and our sample consisted of his readers, the sample was fairly representative of the general fiction-reading public. This and the remaining aspects of our study mentioned above are important in that while the attitudinal impact of literary fiction has been widely assumed both by scholars and by writers and policy makers, the sound, ecologically valid evidence for it is still scarce [29]. In demonstrating that when exposed to a fictional narrative readers can immediately react with raised concern for the well-being of animals our study is a step toward providing such evidence.

In trying to provide this evidence we are admittedly aware of the ethical questions which may be raised about using fiction for such purposes and which have been the subject of much scholarly debate [29]. For instance, it might be argued that the effect observed in our study further confirms the worries expressed by various philosophers and psychologists about the way being exposed to stories of individual suffering can skew our moral judgments [12,30,31]. It could be argued that merely having learned about the plight of one (fictional) monkey is insufficient reason to change how one thinks about (real) whales and whalers (see Table 1, item 1), or about whether “The suffering of animals is an acceptable price for inventing drugs for humans,” as another item on our scale put it. Yet this is precisely what was observed in our experiment.

It could also be argued that using a piece of fiction in the way we propose is manipulative, and that an ethically proper, non-manipulative way to change attitudes toward animals would be through engaging in rational argumentation where one’s purpose in offering such arguments is made explicit. On the other hand, the practice of using literary fiction to try to change attitudes has been widely accepted, even venerated, in recent history and in some cases appears to have led to undeniably desirable social outcomes. Harriet Beecher Stowe’s novel *Uncle Tom’s Cabin*, for instance, is widely acknowledged to have contributed to the abolition of slavery through its portrayals of the plight of African Americans [32]. Such examples suggest that the potential good that could be achieved with the help of the attitudinal impact of literary fiction may outweigh whatever is morally questionable with using literary fiction for that purpose.

Moreover, there is a growing body of research in philosophy and experimental psychology which argues that moral attitudes (including attitudes toward the welfare of others) are based on basic, implicit intuitions that are highly resistant to being changed through moral argument [33,34]. If the results of this research hold then this further strengthens the moral case for using fiction instead of explicit argumentation to make people more concerned about the welfare of animals.

Nonetheless, further work still needs to be done before it can be concluded beyond doubt that the standard forms of campaigning should be supplemented on a large scale by strategies involving literary fiction so far as effecting lasting change on the public’s attitudes toward animal welfare in concerned. What is needed in particular are experiments that would examine the exact mechanism behind the effect we observed and the long-term, as opposed to immediate, effects of fictional narratives such as that of Krajewski’s. Another important direction for further research would be to address what is perhaps the main limitation of our study, namely, that the effect we observed might be due to priming. Admittedly, we cannot preclude that the story itself made the items about animals in the questionnaire more salient to the subjects.

It should also be stressed here that our experiment does not demonstrate that all fictional texts depicting the plight of an animal will have an impact on readers’ attitudes toward animals. Works of fiction can vary greatly in terms of the particular individuals and circumstances they describe. Such differences may contribute to the effect a particular text might have on readers. For instance, consistent with those cases that have been historically acknowledged in which a work of fiction has influenced social attitudes towards animal welfare (such as *Black Beauty*), we used a text in which harm was done to an animal by characters depicted in a negative way. But a fictional text depicting harm being done to an animal by positively depicted protagonists might not yield the same effect as that observed in our study. Similarly, the animal protagonist in the story was a monkey, and had we used a different animal in this capacity, an insect for example, the same effect might not have been obtained.

However, the point of our experiment was not to show that any fictional text will have an impact on attitudes toward animals, but rather that a particular fictional text can do so. This is all that we claim to have achieved. Finally, combined with what we already know about the longitudinal effects of fiction reading in general [10,13,35–38] and about the aforementioned problematic aspects of standard forms of campaigning for animal welfare, the results of our study strongly suggest that the possibility of influencing the public’s concern for animals through literary fiction is worth exploring further.

## Supporting Information

S1 Text Quiz announcements. Announcement A in S1 Text provides an English translation of the original Polish text of the announcement posted on the author’s Facebook profile. Announcement B in S1 Text provides the original Polish version. Announcement C in S1 Text provides an English translation of the original Polish text of the announcement posted on the publisher’s Facebook profile. Announcement D in S1 Text provides the original Polish version.
(DOCX)

S2 Text Experimental narrative used in the study. Narrative A in S2 Text provides an English translation of the original Polish experimental narrative used in the study. Narrative B in S2 Text B provides the original Polish version.
(DOCX)

S3 Text Control narrative used in the study. Narrative A in S3 Text provides an English translation of the original Polish control narrative used in the study. Narrative B in S3 Text provides the original Polish version.
(DOCX)

S4 Text Questionnaire used in the study. Questionnaire A in S4 Text provides an English translation of the original Polish questionnaire used in the study. Questionnaire B in S4 Text B provides the original Polish version.
(DOCX)

S5 Text ATAW Scale. Scale A in S5 Text provides an English translation of the original Polish scale used in the study. Scale B in S5 Text B provides the original Polish version.
(DOCX)
