# Correction: Literary Fiction Influences Attitudes Toward Animal Welfare

**DOI:** 10.1371/journal.pone.0172328

**Published:** 2017-02-10

**Authors:** The PLOS ONE Staff

The Data Availability statement for this paper is incorrect. The correct statement is: The data have been deposited with figshare: http://dx.doi.org/10.6084/m9.figshare.1547933. The publisher apologizes for the error.
