# Supplementary material for: Literary Fiction Influences Attitudes Toward Animal Welfare
Source: PLoS One. 2016 Dec 22;11(12):e0168695. doi: 10.1371/journal.pone.0168695 (PMC5179074; doi:10.1371/journal.pone.0168695)
Supplement: S1 Text — Announcement A in S1 Text provides an English translation of the original Polish text of the announcement posted on the author’s Facebook profile. Announcement B in S1 Text provides the original Polish version. Announcement C in S1 Text provides an English translation of the original Polish text of the announcement posted on the publisher’s Facebook profile. Announcement D in S1 Text provides the original Polish version. (DOCX) [file pone.0168695.s001.docx]

**S1 Text: Quiz announcements**

**Announcement A: an English translation of the original Polish text of the announcement posted on the author’s Facebook profile.**

Dear Sirs and Madams, I would like to invite you to an interesting quiz, which will give you the opportunity to read an unpublished fragment of my latest novel *The Lord of the Numbers* and a chance to win a copy of the book. I am currently cooperating with scholars who would like to study the psychological profile of the readers of my novels by using an internet questionnaire. In order to be able to win a copy, you only have to fill out the questionnaire and answer a quiz question. 77 copies of *The Lord of the Numbers* are waiting for the winners! The questionnaire and further information can be found at the website <http://badanie-czytelnikow.imas.pl/#welcome> [no longer active] All are invited! Marek Krajewski.

**Announcement B: the original Polish version of the announcement posted on the author’s Facebook profile.**

Szanowni Państwo, chciałbym Państwa zaprosić do ciekawej zabawy, dzięki której będziecie Państwo mogli przeczytać przedpremierowy fragment mojej najnowszej powieści pt. *Władca Liczb*, a także uzyskać szansę wygrania tej książki. Współpracuję obecnie z naukowcami, którzy chcieliby zbadać przy pomocy ankiety internetowej, jaki jest profil psychologiczny czytelników moich powieści. Aby móc wygrać książkę, wystarczy tylko wypełnić tę ankietę i odpowiedzieć na pytanie konkursowe. Na zwycięzców czeka 77 egzemplarzy „Władcy Liczb”! Ankietę oraz dalsze informacje znajdziecie Państwo na stronie [http://badanie-czytelnikow.imas.pl/#welcome](http://badanie-czytelnikow.imas.pl/#welcome)  Zapraszam serdecznie! Marek Krajewski.

**Announcement C: an English translation of the original Polish text of the announcement posted on the publisher’s Facebook profile.**

Fancy a copy of the latest Book by Marek Krajewski *The Lord of the Numbers*? If so, then now is the only chance to win it – before the official premiere! 77 copies to grab! You just have to take part in an interesting research project – fill out a questionnaire and answer a quiz question. For more information, follow this link -- > <http://badanie-czytelnikow.imas.pl/>. All are invited! :) ZNAK [the name of the Publisher]

**Announcement D:** **the original Polish text of the announcement posted on the publisher’s Facebook profile.**

Macie ochotę na najnowszą książkę Marka Krajewskiego, *Władca liczb*? ;) Jeśli tak, tylko teraz jedyna szansa, żeby ją wygrać - jeszcze przed oficjalną premierą! Do zgarnięcia aż 77 egzemplarzy! :) Wystarczy, że weźmiecie udział w ciekawym projekcie badawczym - wypełnicie ankietę internetową i odpowiecie na pytanie konkursowe. Więcej informacji w tym linku -- > [http://badanie-czytelnikow.imas.pl/](http://badanie-czytelnikow.imas.pl/). Serdecznie zapraszamy! :) ZNAK
