# Supplementary material for: Literary Fiction Influences Attitudes Toward Animal Welfare
Source: PLoS One. 2016 Dec 22;11(12):e0168695. doi: 10.1371/journal.pone.0168695 (PMC5179074; doi:10.1371/journal.pone.0168695)
Supplement: S2 Text — Narrative A in S2 Text provides an English translation of the original Polish experimental narrative used in the study. Narrative B in S2 Text B provides the original Polish version. (DOCX) [file pone.0168695.s002.docx]

**S2 Text. Experimental narrative used in the study**

**Narrative A: an English translation of the original Polish experimental narrative used in the study.**

That I myself was not a follower of Belmispar did not mean that our city was devoid of his acolytes. While I was introducing Leocadie to Zaranek-Plater’s mathematical demonology, in one of Wrocław’s apartments a likely follower of the Lord of the Numbers was conducting his experiments.

The object of his studies was a monkey named Clotho as a tribute to one of the mythical weavers, who – together with her sisters Lachesis and Atropos – weaved the thread of human existence.

Fifteen years back, that black-and-white capuchin monkey lived in a Venezuelan jungle. The warmth of her mother’s belly, to which she clung tightly, filled her with a sense of security when her mother jumped from one woody balsa treetop to another during her air travels. On one of such trips, the monkey felt the muscles of her mother’s belly contracting violently. The feeling was unpleasant, and so was the one following it, when the little creature felt the power of gravity for the first time in her life. She turned her head around and did not see the usual sight – tree leaves moving below her. Instead, she noticed brown hairless skin and hard heels stepping firmly on the decomposing undergrowth of the jungle. Intense anxiety made her let go off her mother’s belly. She slid down, but did not fall. This was prevented by a net tightly knit from a liana.

On that day, the monkey learned her first lesson about the pain of separation. A native from the tribe of Warao sold her mother to a European trader, and she herself was given to the native’s children as a toy. The children would sometimes hug and stroke her, and pricked and pinched her on other times. When, in an act of self-defense, she bit the native’s beloved son on his finger, her fate was sealed. First, she was painfully kicked around the house, and then she found herself in the cargo hold of a Dutch freighter going to Amsterdam.

Thus began the true toil of her simian existence. She was sold to an animal wholesaler who then sold her to a travelling circus. There she discovered a new kind of pain – not the slight one which she once felt as a result of venomous ant bites or the pinches, and even kicks, of the native children. No, the new pain was overwhelming and expansive. Its effects were not confined to the particular place on her tiny body touched by the instrument that inflicted suffering: it was spreading constantly and in irregular waves.

The Circus trainer was a chain smoker and he liked it that he could do at work what he liked best. First, when the monkey refused to roller skate and walk on stilts, he pulled her by the chain attached to her neck. The animal would fall to the sand of the arena and screamed in fear. Unfortunately, it would then repeat her mistakes and throw away with aversion the many toys and objects it was supposed to use for the amusement of the circus audience. Faced with such failures, it dawned on the circus trainer that he could use a cigarette. He grabbed the monkey by her throat with one hand, and pulled a hood on her head with the other. Convinced that, as a result, the animal would be unable to bite him, he then took a cigarette out of his mouth and pressed it against a tiny heel.

The penetrating shriek of the animal spanned a few registers. The monkey’s body, wrapped in an enormous hood, trembled spasmodically, and her nervous system reacted by relaxing sphincters.

The trainer withdrew his hand in disgust, grabbed the creature by its hand, dragged the tiny, still trembling body through the sand of the arena, and then, having waited for an hour, he would scrape off the dust covered muck.

The tiny animal was a fast learner. No more than a week had to pass until the trainer did not have to strain himself anymore and could use cigarettes according to their usual purpose. After a few burns the monkey became obedient. Consistent with the findings of Ivan Pavlov, she associated pain with the darkness of the hood, not with the glow of the cigarette. Whenever darkness would fall upon her, out of fear, she would bite the soft felt of the hood. Eventually, its mere sight was enough to make her do what was wanted of her. At night, when it would become pitch black, the monkey would go insane and bite everything around her.

Soon she became an attraction of the travelling circus. She let herself be stroked and fed. She even learned how to shake hands with the audience. One day the circus found itself in the distant Poland, where it amused German soldiers. In Cracow, she became the darling of the daughter of a certain general. During one of the performances, the child was encouraged by a clown to shake hands with the animal. She did so and precisely at this moment all lights went out. It was obvious for the monkey that the hood had once again separated her from light. She reacted the way she usually did when it would become dark. She bared her teeth and made use of them.

On the very same night, the monkey was sold for pennies to an organ grinder, who did not want anything from her apart from sitting on his organ. He looked after her with such care that he did not even economize on lamp oil and put a lit lamp next to her cage at night. Unfortunately, that *dolce vita*^[[1]](#footnote-1)^ did not last long. The organ grinder passed away, and the monkey, christened Clotho by her new owner, only then encountered the true reality of pain.

This one was the most terrifying the animal ever felt. It twisted and paralyzed her body for a few seconds. Its source was neither a particular point or area on her body – the pain now resided at the very center of the capuchin, it wrenched her with spasms and threw her against the walls of the cage.

The worst thing was that she could do nothing about it. The man would put inside the cage an iron stand with two ladders leading to a small platform. One of them was black, the other white. Lying on the platform, there was a walnut. The creature would happily climb for the walnut – using either the white or the black ladder. Then the man would draw out two protruding wires in her direction. Electricity would twist her body and force a high-pitched shriek out of the tiny throat. The man would smile friendly, say something in a silent voice and touch one or the other ladder with a pointer – the white and the black one, in turns. Clotho did not know what was on her tormentor’s mind. Afraid of the wires, she jumped from one ladder to the other like crazy, blindly. Then the man would apply electric shocks again. Apparently, he demanded something else. She did not understand that he wanted to make her disorderly jumps less chaotic – that all that he wanted was that she first climbed the black ladder, and then immediately the white one.

Clotho failed to grasp the man’s intentions. She was helpless. All she could do was to look into the eyes of the tormentor approaching her. And then to suffer.

**Narrative B: the original Polish version of the experimental narrative used in the study.**

To, że nie byłem wyznawcą Belmispara, nie znaczyło, że innych jego akolitów nie ma w naszym mieście. W tym czasie, gdy zapoznawałem Leokadię z matematyczną demonologią Zaranek-Platera, w jednym z wrocławskich mieszkań prowadził swe eksperymenty najpewniej jakiś czciciel władcy liczb.

Przedmiotem jego badań była małpka nosząca imię Kloto – na cześć jednej z mitycznych prządek, która obok swych sióstr – Lachesis i Atropos – przędła nić ludzkiego żywota.

Ta czarno-biała kapucynka piętnaście lat wcześniej żyła w wenezuelskiej dżungli. Znane dobrze ciepło matczynego brzucha, którego się kurczowo trzymała, napełniało ją poczuciem bezpieczeństwa w czasie licznych napowietrznych podróży, jakie jej matka sobie urządzała, skacząc po rozłożystych koronach drzew balsa. W czasie jednego z takich wojaży małpka poczuła, iż mięśnie brzucha jej matki kurczą się gwałtownie. To uczucie nie było przyjemne, podobnie jak następne – kiedy mała istota po raz pierwszy poznała siłę grawitacji. Wykręciła głowę i nie ujrzała zwykłego widoku – przesuwających się pod nią liści drzew. Zamiast nich widziała teraz brunatną bezwłosą skórę i twarde pięty stąpające mocno po gnijącym poszyciu dżungli. Silny niepokój sprawił, że małpka puściła brzuch matki. Obsunęła się nieco, ale nie upadła. Uniemożliwiła jej to gęsta siatka upleciona z liany.

Tego dnia małpka poznała gorycz rozstania. Indianin z plemienia Warao sprzedał jej matkę europejskiemu handlarzowi, a ją samą oddał swoim dzieciom do zabawy. Dzieci czasem ją tuliły i głaskały, czasem kłuły i szczypały. Kiedy w samoobronie ugryzła w palec ukochanego synka Indianina, jej los został przypieczętowany. Najpierw wymierzono jej kilka bolesnych kopniaków, po których fruwała po chacie, po czym znalazła się w ładowni holenderskiego frachtowca, który *via* Gujana popłynął do Amsterdamu.

Wtedy zaczął się prawdziwy znój jej małpiego życia. Została najpierw sprzedana hurtownikowi zwierząt, który z kolei odsprzedał ją objazdowemu cyrkowi. Tam po raz pierwszy małpka poznała ból – nie ten lekki, który niegdyś wywoływały ukąszenia jadowitych mrówek czy uszczypnięcia, a nawet kopniaki indiańskich dzieci. Nie, ten nowy ból był przejmujący i ekspansywny. Swym działaniem obejmował nie tylko to miejsce drobnego ciała, do którego przylgnęło narzędzie wywołujące cierpienie: on się wciąż rozszerzał w nieregularnych falach.

Cyrkowy treser był nałogowym palaczem i bardzo mu się podobało, iż przy swej robocie robi to, co najbardziej lubi. Najpierw, kiedy małpka nie chciała jeździć na wrotkach ani chodzić na szczudłach, ciągnął ją za łańcuch, który miała uwiązany do szyi. Zwierzę upadało w piach areny i krzyczało ze strachu. Potem niestety powtarzało swoje błędy i z niechęcią odrzucało kolejne zabawki i przedmioty, których miało używać ku uciesze cyrkowej gawiedzi. Wobec swych treserskich porażek cyrkowiec wpadł na pomysł użycia papierosa. Jedną ręką chwycił małpkę za gardło, drugą zaś nałożył jej kaptur na głowę. Wtedy, mając pewność, że zwierzątko go nie pokąsa, wyjął papierosa z ust i przyłożył go do malutkiej pięty.

Przenikliwy pisk zwierzęcia objął kilka rejestrów, ciało małpki spętane ogromnym kapturem zadrżało spazmatycznie, układ przywspółczulny zareagował poluzowaniem zwieraczy.

Treser cofnął ze wstrętem rękę, chwycił stworzenie za łapę, wytarzał małe, wciąż drżące ciało w piachu areny, po czym poczekał i po godzinie zdrapał skrobaczką oprószone pyłem łajno.

Zwierzątko było teraz coraz bardziej pojętne. Po tygodniu treser nie musiał się męczyć i mógł wykorzystywać papierosy wyłącznie zgodnie z ich przeznaczeniem. Po kilku bowiem przypaleniach małpka była posłuszna. Zgodnie z ustaleniami Iwana Pawłowa ból kojarzyła z ciemnością wywołaną kapturem, nie z żarem papierosa. Kiedy zatem opadał na nią mrok, gryzła ze strachu miękki filc kaptura. Potem wystarczał już sam jego widok, a małpka robiła wszystko, czego od niej żądano. W nocy, kiedy zapadał całkowity mrok, małpka szalała i gryzła wszystko dokoła.

Szybko stała się atrakcją wędrownego cyrku. Pozwalała się głaskać i karmić, nauczyła się nawet podawać łapę. Pewnego dnia cyrk zawędrował do dalekiej Polski, gdzie umilał czas niemieckim żołnierzom. W mieście Krakowie przypadła bardzo do gustu córeczce pewnego generała. W czasie występu cyrkowego dziecko zostało poproszone przez klauna o podanie zwierzątku ręki. Zrobiło to i w tym momencie zapadła ciemność. Dla małpki było oczywiste, że kaptur po raz kolejny odciął ją od światła. Zareagowała tak jak zawsze, gdy robiło się ciemno. Odsłoniła ostre zęby i zrobiła z nich użytek.

Tego samego wieczoru kapucynka została sprzedana za grosze kataryniarzowi, który niczego od niej nie wymagał oprócz tego, by siedziała na jego katarynce. Dbał o nią tak troskliwie, że nie oszczędzał na nafcie i w nocy stawiał obok jej klatki zapaloną lampę. Niestety *dolce vita*^[[2]](#footnote-2)^ nie trwała długo. Kataryniarz zmarł, a małpka, obdarzona przez nowego właściciela imieniem Kloto, dopiero teraz poznała, co to znaczy prawdziwy ból.

Był on najstraszniejszy, jaki kiedykolwiek to stworzenie czuło. Wykrzywiał i unieruchamiał przez kilka sekund całe ciało. Jego źródłem nie był punkt ani obszar – ból siedział teraz w samym środku kapucynki, szarpał ciągłym spazmem, wyciskał z niej ekskrementy i rzucał nią o ściany klatki.

Najgorzej, że nie można mu było nijak zaradzić. Człowiek wkładał od góry do klatki żelazny stojak z dwiema drabinkami, które prowadziły na mały podest. Jedna z nich była czarna, druga biała. Na podeście był orzech włoski. Stworzenie cieszyło się i wspinało się po orzech – po białej lub po czarnej drabince. Wtedy człowiek wyciągał ku niej dwa wystające druty. Prąd wykręcał jej ciało i wyduszał z małego gardła wysoki pisk. Człowiek uśmiechał się przyjaźnie, mówił coś cicho i wskaźnikiem uderzał to w jedną, to w drugą drabinkę. Na przemian – raz w białą, raz w czarną. Kloto nie wiedziała, o co chodzi jej oprawcy. Bojąc się drutów, skakała po drabinkach jak szalona, na oślep. Wtedy człowiek raził ją prądem. Najwyraźniej żądał czegoś innego. Nie rozumiała, że chciał jej bezładne skoki pozbawić chaotyczności – że pragnął tylko tego, by wspinała się po orzech najpierw po czarnej, a zaraz potem po białej drabince.

Kloto nie rozumiała intencyj człowieka. Była bezradna. Mogła tylko patrzeć w oczy zbliżającego się do niej oprawcy. A potem już tylko cierpieć.

1. *Dolce vita* (Italian) – sweet life. [↑](#footnote-ref-1)
2. *dolce vita* (wł.) – słodkie życie. [↑](#footnote-ref-2)
