# Supplementary material for: Literary Fiction Influences Attitudes Toward Animal Welfare
Source: PLoS One. 2016 Dec 22;11(12):e0168695. doi: 10.1371/journal.pone.0168695 (PMC5179074; doi:10.1371/journal.pone.0168695)
Supplement: S3 Text — Narrative A in S3 Text provides an English translation of the original Polish control narrative used in the study. Narrative B in S3 Text provides the original Polish version. (DOCX) [file pone.0168695.s003.docx]

**S3 Text. Control narrative used in the study**

**Narrative A: an English translation of the original Polish control narrative used in the study.**

The man standing just outside my garden kept his eye on me constantly. When I took a look at him, he raised his bowler hat, revealing a few streaks of hair stuck firmly to his bald skull. I responded with a similar gesture, the only difference being that my head was entirely bald as I had nothing but utter scorn for all kinds of comb-overs. The heat of July morning obviously made no impression on the corpulent gentleman since he was dressed in an unusually hermetic way, so to speak. He wore a brown three-piece suit made of wool and a bowler hat of the same color. A bow tie of a slightly lighter shade of brown sealed his outfit under his chin, preventing any influx of fresh air from above. In his browns, he looked like an English lord who by some strange twist of fate had been transported to the white-hot Wrocław from his moorlands in Yorkshire.

– Do I have the honor of speaking with Mr. Edward Popielski? – he asked.

– Yes indeed, it’s me. – I replied.

I scanned the man from head to toes, and could not resist bursting with laughter upon seeing his Georgian elegance, which was complemented by knicker-bockers, the bottoms of which were tucked in long checkered gaiters.

– I have no idea what exactly you find so funny about me, but I wanted to assure you I’m not a figure from a cabaret, Mr. Popielski. – The man, who on first look seemed to be a sexagenarian, raised his voice – I am Count Władysław Zaranek-Plater.

He raised his eyes to the sky. Apparently, he was expecting an apology and an expression of reverence, but what he got was something else entirely.

– I’m about to star cheering – I smiled broadly – And then I’ll ask you to sign my friendship book…

That joke apparently made a bigger impression on him than the heat, because he took off the bowler-hat and wiped sweat of his forehead with a large checkered handkerchief, going through amazing contortions so as not to ruin his elaborate hairdo.

– I am a friend of your boss, Aleksander Beck. – He handed me his name card and wheezed scornfully, putting his bowler-hat back on his head. – I know what you really do for a living and I wanted to hire you to do a certain important and exceptionally well-paid job.

The adverb “really” put me out of the mood for making jokes. My stint as a “legal representative” at Beck’s law firm, which had lasted four years already, was no mystery and my daily job no cause for sensation. If anyone had followed me, they would have seen that every day at eight in the morning I would come to the office at Świerczewskiego Street, browse the files of current cases and make some notes. If that hypothetical investigator had felt like observing me a bit longer, then he would have learned that I met with various people and that I just talked to them.

What I “really” did, however, was not that far from my previous job as a detective. For the so-called gathering of evidence consisted in looking for vices and weaknesses of the prosecution witnesses and then skillfully using those flaws. Since this activity often involved a bit of delicate blackmail – if not intimidation – I preferred that third parties, such as this Count straight out of an operetta, did not know what my job “really” consists in.

– Please do not bother people in the street – I said in a calm voice – If you are a salesman, sell your whetstones to somebody else.

With a volume of Cicero tucked under my arm, I walked off toward the ruins at the corner of Górnickiego and Benedektyńska street. The cathedral towers were gleaming in the distance.

Count Zaranek-Plater was running after me.

– Please wait for me, Mr. Popielski. – He breathed heavily, and the tone of his voice seemed pleading to me. – There’s a cab waiting for us just around the corner. We can go to Mr. Beck, and he will confirm everything: that I talked to him about you, and that he recommended you to me… At this very moment he is on the beach by Odra with his family, but he will not be upset if we disturb him … He is an old friend, a compatriot, we come from the same place.

I stopped. I knew that on that Sunday Beck was in fact going with his wife and two children to the beach by Odra. Just yesterday, his beautiful wife visited our office and – disturbing with her graceful moves the peace of my young assistant – she interrogated me thoroughly (as if I had been some kind of expert on water engineering!) about the dangers lurking for those bathing in the vicinity of the Opatowicka Island.

– All right. – I watched with amusement as Zaranek-Plater tried to evenly spread the streaks of hair on his skull. – I believe your Lordship… Well … I will not invite you into my house as my cousin is giving lessons at the moment. I suggest that we take a walk in the Botanical Garden. This is where I have just been heading and this is also where your Lordship will present his job order to me. So? Shall we take a walk?

– We will take a ride! This is a hired driver. – The count replied with confidence and pointed toward a cab parked on the corner of Ładna and Miła, with the driver standing next to it, sporting a wife-beater and a beret with a stem on top. And we will not got to the Botanical Garden, but to the Main Train Station.

– What for? Do you want to take me out on a suburban trip?

– I lead a very regular life! Eleven o’clock is fast approaching and at this time of the day I always eat a cream-filled biscuit and drink coffee at the Main Train Station café… I invite you to a biscuit!

I felt that I, too, was beginning to be affected by the July heat. I took off my hat, unbuttoned another button of my shirt, whose collar I had previously laid out on the shoulders of my jacket.

– Your Lordship – I waved myself with the hat. – I suffer greatly when I am forced to waste my time. And I do not like to suffer. How can I know that I will like your biscuit? I am not looking for an extra pay.

– Mr. Popielski – Zaranek-Plater took me by the elbow and started to lead gently toward the cab. – If you reject my offer, I will give you five hundred for the trouble and the cab will drive you back home.

– And if I take it?

– Then my friend Olek Beck will curse me because he will lose a reliable employee!

– Could your Lordship be a bit clearer? – Now it was my turn to wipe sweat off my bald scalp. – It is too hot for conditionals without consequents. What will happen if I take your job offer? What will happen, my Lord? Finish that sentence!

– You will not have to work until the end of your life – Said the count lazily and whispered a very large and tempting sum right into my ear – So what do you think? Will you take the risk and spend a moment in my company? The worst case scenario is that you will then show the five hundred to your cousin and say: “I’m sorry I’m late for lunch. I wasted some time with a certain eccentric!” I nodded my head, and after a short while, I was rocking on the back seat of a Warszawa and looking with disgust at the gray-bristle covered neck of the cab driver.

**Narrative B: the original Polish version of the control narrative used in the study**

Mężczyzna stojący koło mojego ogródka nie spuszczał ze mnie wzroku. Kiedy na niego spojrzałem, uchylił melonika, ukazując kilka pasm włosów przyklejonych do łysej czaszki. Odwzajemniłem mu się tym samym gestem, z tą wszakże różnicą, że moja głowa była całkiem łysa, gdyż wszelkie fryzjerskie zaczeski i pożyczki miałem zawsze w głębokiej pogardzie. Na niewysokim, dość korpulentnym panu ciepło lipcowego poranka nie robiło najwyraźniej większego wrażenia, bo był ubrany nadzwyczaj – by tak rzec – szczelnie. Miał mianowicie na sobie wełniany brązowy garnitur z kamizelką i takiejż barwy melonik. Nieco jaśniejsza ręcznie wiązana mucha uszczelniała go pod szyją i od góry uniemożliwiała dopływ świeżego powietrza. W swych brązach wyglądał jak angielski lord, który dziwnym zrządzeniem losu żywcem został przeniesiony do rozpalonego upałem Wrocławia ze swych wrzosowisk w Yorkshire.

– Czy mam zaszczyt z panem Edwardem Popielskim? – zapytał.

– Tak, owszem, to ja – odrzekłem.

Obejrzałem przybysza od stóp do głów i nie mogłem się opanować, by nie parsknąć śmiechem na widok jego przedwojennej elegancji, która była godnie dopełniona przez pumpy, wpuszczone w długie kraciaste getry.

– Nie wiem, co pana tak śmieszy, lecz ja nie jestem postacią z singiel-tanglu, panie Popielski. – Sześćdziesięcioletni na oko mężczyzna podniósł głos. – Jestem Władysław hrabia Zaranek-Plater.

Wzniósł oczy ku górze. Najwidoczniej spodziewał się przeprosin i podziwu, ale doczekał się czegoś zupełnie innego.

– Zaraz zacznę wiwatować! – Uśmiechnąłem się szeroko. – A potem poproszę, by się pan wpisał do mojego sztambucha...

Dowcip ten najwyraźniej zrobił na przybyszu większe wrażenie niż upał, bo zdjął melonik i wielką kraciastą chustą otarł pot, zręcznie przy tym manewrując, by nie zburzyć swej misternej fryzury.

– Jestem przyjacielem pańskiego szefa mecenasa Aleksandra Becka. – Wręczył mi swoją wizytówkę i prychnął pogardliwie, włożywszy na powrót melonik. – Wiem, czym się pan naprawdę zajmuje, i chcę pana wynająć do ważnego, nadzwyczaj dobrze płatnego zadania.

Przysłówek „naprawdę” sprawił, że straciłem ochotę do dalszych żartów. Moje czteroletnie już zatrudnienie u mecenasa Becka jako „pełnomocnika procesowego” nie było żadną tajemnicą, a moja codzienna praca żadną sensacją. Gdyby ktoś mnie śledził, zobaczyłby, że dzień w dzień o ósmej rano przychodzę do biura mecenasa na Świerczewskiego, przeglądam akta aktualnych spraw i sporządzam jakieś notatki. Gdyby temu hipotetycznemu śledzącemu chciało się jeszcze dłużej mnie poobserwować, to dowiedziałby się, że spotykam się z różnymi ludźmi i po prostu z nimi rozmawiam.

„Naprawdę” jednak zajmowałem się czymś, co nie było wcale odległe od mej dawnej detektywistycznej profesji. Tak zwane zbieranie materiału procesowego polegało bowiem głównie na szukaniu jakichś wad i ułomności świadków oskarżenia, a potem na umiejętnym wykorzystywaniu tych skaz. Ponieważ ta działalność często była połączona z delikatnym szantażem – by nie rzec zastraszaniem – niespecjalnie mi zależało na tym, aby osoby postronne, takie jak na przykład ów operetkowy hrabia, wiedziały, na czym „naprawdę” polega moja praca.

– Proszę nie zaczepiać ludzi na ulicy – powiedziałem spokojnie. – Jeśli pan jest komiwojażerem, to niech pan komu innemu sprzedaje swoje magiczne osełki.

Z Cyceronem pod pachą poszedłem w stronę ruin na rogu Górnickiego i Benedyktyńskiej. W oddali błyszczały w słońcu katedralne wieże.

Hrabia Zaranek-Plater biegł za mną.

– Niechże pan poczeka, panie Popielski. – Ciężko dyszał, a ton jego głosu zdał mi się błagalny. – Tu zaraz jest taksówka. Możemy pojechać do mecenasa Becka i on wszystko potwierdzi... Że z nim rozmawiałem o panu, że mi pana polecił... On jest teraz nad Odrą z rodziną, ale wcale się nie pogniewa, jeśli mu przeszkodzimy... To mój stary przyjaciel, krajan, pochodzimy z tych samych stron...

Przystanąłem. Rzeczywiście wiedziałem o tym, że Beck wybiera się w tę niedzielę ze swoją żoną i z dwojgiem dzieci na nadodrzańską plażę za Biskupinem. Jeszcze wczoraj piękna mecenasowa, zawitała do naszej kancelarii i – burząc swymi ruchami spokój młodego aplikanta – wypytywała mnie dokładnie (jakbym był co najmniej znawcą urządzeń wodnych!) o niebezpieczeństwa, jakie czyhają na kąpiących się koło Wyspy Opatowickiej.

– Dobrze. – Patrzyłem z rozbawieniem, jak Zaranek-Plater rozstawia teraz w równej odległości pasma włosów na swej czaszce. – Wierzę panu hrabiemu... No cóż... Nie zapraszam teraz do domu, bo tam moja kuzynka udziela lekcyj. Proponuję, abyśmy pospacerowali po Ogrodzie Botanicznym. Tam właśnie szedłem i tam mi pan hrabia przedstawi swoje zlecenie. To co? Idziemy?

– Jedziemy! To wynajęty szofer! – odparł pewnie hrabia i wskazał na taksówkę na rogu Ładnej i Miłej, obok której stał kierowca ubrany w podkoszulek i beret z antenką. – I pojedziemy nie do Ogrodu Botanicznego, ale na Dworzec Główny.

– Po co? Chce się pan wybrać ze mną na podmiejską wycieczkę?

– Mój tryb życia jest bardzo regularny! Oto zbliża się jedenasta, a ja o tej porze zawsze jem ciastko z kremem i piję kawę w kawiarni dworcowej... No, zapraszam pana na ciastko!

Poczułem, że i na mnie lipcowy upał zaczął oddziaływać. Zdjąłem kapelusz i rozpiąłem drugi guzik koszuli, której kołnierz wyłożyłem już wcześniej na marynarkę.

– Panie hrabio – powachlowałem się kapeluszem – ja bardzo cierpię, kiedy tracę czas. A nie lubię cierpieć. Skąd ja wiem, że pańskie ciastko mi posmakuje? Ja nie szukam wcale dodatkowego zarobku...

– Panie Popielski – Zaranek-Plater ujął mnie pod łokieć i zaczął delikatnie prowadzić w stronę taksówki –Jeśli pan odrzuci moją propozycję, zapłacę panu pięć setek za fatygę i taksówka odwiezie pana do domu...

– A jeśli ją przyjmę?

– To wtedy mój przyjaciel Olek Beck mnie przeklnie, bo straci niezawodnego pracownika!

– Jaśniej, proszę pana. – Tym razem ja wytarłem pot z łysiny. – Jest za gorąco na implikacje bez następnika. Jeśli przyjmę pańskie zlecenie, to co się stanie? Co wtedy będzie, panie ładny? Dokończ pan!

– Nie będzie pan musiał pracować do końca życia – powiedział leniwie hrabia i wyszeptał mi do ucha bardzo dużą i kuszącą kwotę. – No to co? Zaryzykuje pan i spędzi trochę czasu w moim towarzystwie? Najwyżej pokaże pan później kuzynce pięćset złotych i powie pan: „Przepraszam, że spóźniłem się na obiad, zmitrężyłem trochę czasu z pewnym ekscentrykiem”!

Kiwnąłem głową, a po chwili kołysałem się na tylnym siedzeniu warszawy i patrzyłem ze wstrętem w zarośnięty siwą szczeciną kark taksówkarza.
