# Supplementary material for: Literary Fiction Influences Attitudes Toward Animal Welfare
Source: PLoS One. 2016 Dec 22;11(12):e0168695. doi: 10.1371/journal.pone.0168695 (PMC5179074; doi:10.1371/journal.pone.0168695)
Supplement: S4 Text — Questionnaire A in S4 Text provides an English translation of the original Polish questionnaire used in the study. Questionnaire B in S4 Text B provides the original Polish version. (DOCX) [file pone.0168695.s004.docx]

**S4 Text. Questionnaire used in the study**

**Questionnaire A: an English translation of the original Polish questionnaire used in the study.**

*Intro*

Study of the psychological profile of Marek Krajewski’s readers

Thank you for your interest in taking part in our study. The Questionnaire consists of several parts. First, you will read a fragment of the latest book by the author, and then we will ask you a series of questions. We hope that filling out the questionnaire will be an interesting experience for you.

The questionnaire is anonymous. All answers and data gathered are confidential. They will be used collectively and only for research purposes.

The study should take ca. 20-25 minutes.

**FRAGMENT OF THE BOOK**

Read closely the following fragment of the latest book by Marek Krajewski. Then turn to the questionnaire. Some of the questions will concern your general views, others your impressions about the text.

[Participants were then randomly assigned either to Experimental Narrative or Control Narrative]

**MAIN PART**

The purpose of this study is to study the views and attitudes of Marek Krajewski’s readers. You will see a questionnaire consisting of 40 questions. Read closely each of them and mark to what degree you agree with each of them.

There are no good and bad answers here. We will appreciate honest answers.

PS. At one spot we placed a control question, where you will be asked to mark one particular answer indicated by us. Don’t miss it, good luck!

**Scale of answer choices:**

Completely disagree 1

Disagree 2

Somewhat disagree 3

Neither agree nor disagree 4

Somewhat agree 5

Agree 6

Completely agree 7

Page 1

| 1 | I see myself as extraverted, enthusiastic. |
| --- | --- |
| 2 | Education in the humanities gives one as good prospects as education in STEM. |
| 3 | In vitro fertilization is immoral. |
| 4 | I see myself as critical, quarrelsome. |
| 5 | Our country needs a powerful leader, in order to destroy the radical and immoral currents  prevailing in society today. |
| 6 | Genetically modified food should be freely sold in stores. |
| 7 | The slaughter of whales and dolphins should be immediately stopped even if it means that some people will be put out of work |
| 8 | Our country needs free thinkers, who will have the courage to stand up against traditional ways,  even if this upsets many people. |
| 9 | I see myself as dependable, self-disciplined. |
| 10 | The ‘‘old-fashioned ways’’ and ‘‘old-fashioned values’’ still show the best way to live. |

Page 2

| 11 | Our society would be better off if we showed tolerance and understanding for untraditional values and opinions. |
| --- | --- |
| 12 | Polish citizens should have more access to guns than they have today. |
| 13 | The suffering of animals is an acceptable price for inventing drugs for humans. |
| 14 | I see myself as anxious, easily upset. |
| 15 | God's laws about abortion, pornography and marriage must be strictly followed before it is  too late, violations must be punished. |
| 16 | Cultural minorities should be supported and protected. |
| 17 | I support the legalization of marijuana. |
| 18 | Human needs should always come before the needs of animals. |
| 19 | I see myself as open to new experiences, complex. |
| 20 | The society needs to show openness towards people thinking differently, rather than a strong leader, the world is not particularly evil or dangerous. |

Page 3

| 21 | It would be best if newspapers were censored so that people would not be able to get hold of destructive and disgusting material. |
| --- | --- |
| 22 | Many good people challenge the state, criticize the church and ignore ‘‘the normal way of living’’. |
| 23 | I see myself as reserved, quiet. |
| 24 | Our forefathers ought to be honored more for the way they have built our society, at the same time we ought to put an end to those forces destroying it. |
| 25 | The health care system should be privatized. |
| 26 | I feel personally responsible for helping animals in need. |
| 27 | People ought to put less attention to the Bible and religion, instead they ought to develop their own moral standards. |
| • | Control question. Tick „somewhat disagree” |
| 28 | I see myself as sympathetic, warm. |
| 29 | There are many radical, immoral people trying to ruin things; the society ought to stop them. |
| 30 | I would like EURO currency to be introduced in Poland instead of the Polish Złoty. |

Page 4

| 31 | The low costs of food production do not justify maintaining animals under poor conditions. |
| --- | --- |
| 32 | I see myself as disorganized, careless. |
| 33 | It is better to accept bad literature than to censor it. |
| 34 | Facts show that we have to be harder against crime and sexual immorality,  in order to uphold law and order. |
| 35 | Apes should be granted rights similar to human rights. |
| 36 | I see myself as calm, emotionally stable. |
| 37 | The situation in the society of today would be improved if troublemakers were treated with reason and humanity. |
| 38 | I see myself as conventional, uncreative. |
| 39 | Basically, humans have the right to use animals as we see fit. |
| 40 | If the society so wants, it is the duty of every true citizen to help eliminate the evil that poisons our country from within. |

**QUESTIONS CONCERNING THE TEXT**

Now we would like to ask you to answer questions concerning the fragment of Marek Krajewski’s novel you read in the beginning. Please mark the number you think best fits your opinion about the text.

**Scale of answer choices:**

Completely disagree 1

Disagree 2

Somewhat disagree 3

Neither agree nor disagree 4

Somewhat agree 5

Agree 6

Completely agree 7

| Version for the experimental group | |
| --- | --- |
| 1 | While I was reading the narrative, I could easily picture the events in it taking place. |
| 2 | While I was reading the narrative, activity going on in the room around me was on my mind. |
| 3 | I could picture myself in the scene of the events described in the narrative. |
| 4 | I was mentally involved in the narrative while reading it. |
| 5 | After the narrative ended, I found it easy to put it out of my mind. |
| 6 | I wanted to learn how the narrative ended. |
| 7 | The narrative affected me emotionally. |
| 8 | I found myself thinking of ways the narrative could have turned out differently. |
| 9 | I found my mind wandering while reading the narrative. |
| 10 | The events in the narrative are relevant to my everyday life. |
| 11 | The events in the narrative have changed my life. |
| 12 | I had a vivid mental image of the monkey. |
| 13 | I had a vivid mental image of the circus. |

| Version for the control group | |
| --- | --- |
| 1 | While I was reading the narrative, I could easily picture the events in it taking place. |
| 2 | While I was reading the narrative, activity going on in the room around me was on my mind. |
| 3 | I could picture myself in the scene of the events described in the narrative. |
| 4 | I was mentally involved in the narrative while reading it. |
| 5 | After the narrative ended, I found it easy to put it out of my mind. |
| 6 | I wanted to learn how the narrative ended. |
| 7 | The narrative affected me emotionally. |
| 8 | I found myself thinking of ways the narrative could have turned out differently. |
| 9 | I found my mind wandering while reading the narrative. |
| 10 | The events in the narrative are relevant to my everyday life. |
| 11 | The events in the narrative have changed my life. |
| 12 | I had a vivid mental image of the restaurant. |
| 13 | I had a vivid mental image of the taxi driver. |

**DEMOGRAPHIC DATA QUESTIONS**

Thank you for all your answers. Now there are only a few demographic questions left.

**Mark the size of the town you currently live in**

1. Village
2. Town 25 000 and fewer inhabitants
3. Town 25 - 50 tys. inhabitants
4. Town 51 - 100 tys. inhabitants
5. Town 101 - 200 tys. inhabitants
6. City 201 - 500 tys. inhabitants
7. City above 500 000 inhabitants

**What is your educational background**

1. Primary/ junior high/vocational
2. High school/ post-high school
3. Ba./Ma./Ph.D

**Answer yes or no**

|  |  | Yes | No |
| --- | --- | --- | --- |
| 1 | Do you have siblings | 1 | 2 |
| 2 | Do you have a car | 1 | 2 |
| 3 | Do you keep pets | 1 | 2 |
| 4 | Do you exercise regularly | 1 | 2 |
| 5 | Do you abstain from drining alcohol | 1 | 2 |
| 6 | Are you a vegetarian | 1 | 2 |
| 7 | Are you a vegan | 1 | 2 |
| 8 | Is crime fiction your FAVORITE literary genre | 1 | 2 |
| 9 | Are you generally in good health | 1 | 2 |
| 10 | Have you ever suffered any injuries? | 1 | 2 |

**If you have siblings**

**State the age and gender of your siblings**

......................

**If you have a car**

**What brand of car do you have and how long you have had it for?**

......................

**If you have pets**

**Which species and for how long you have had them for?**

......................

**If you exercise regularly**

**What kind of exercises and for how long have been exercising?**

......................

**If you have been injured**

**What was the cause?**

1. Transport accident
2. Chemical substances
3. Mechanical injury
4. Animal attack
5. Other, which? ..............

**QUIZ**

That is all, thank you for your efforts! Now let us have some fun:)

To take part in the quiz, please fill out the form below and answer a quiz question

Your e-mail address: ..............................

Name and address: .........................

[] I hereby confirm that I have read and accept the terms of the quiz „The study of the psychological profile of Marek Krajewski’s readers ” and that I agree to the processing of my personal data contained in the form , by IMAS International Sp. z o.o. Wrocław, ul. Braci Gierymskich 156, only for the purpose and to extend necessary for running the quiz – not longer than for the duration of the quiz and until any potential demands are stale.

The quiz question is:

How many have fell victim to the Lord of Numbers in the book? 5, 6, 8,9, 16, 17?

Thank you for participating in the study!

**Questionnaire B: the original Polish version of the questionnaire used in the study**

*Wstęp*

Badanie profilu czytelników Marka Krajewskiego!

Dziękujemy Ci za chęć uczestnictwa w naszym badaniu. Ankieta składa się z kilku części, w pierwszej z nich przeczytasz fragment najnowszej książki Autora, a następnie zadamy Ci szereg pytań. Mamy nadzieję, że wypełnianie ankiety będzie dla Ciebie ciekawym przeżyciem.

Ankieta jest anonimowa. Wszystkie odpowiedzi oraz dane są poufne i będą wykorzystywane jedynie zbiorczo, w celach badawczych.

Ankieta potrwa ok. 20-25 minut.

**FRAGMENT KSIĄŻKI**

Przeczytaj uważnie fragment najnowszej książki Marka Krajewskiego. Następnie przejdź do kwestionariusza. Część pytań dotyczyć będzie Twoich ogólnych poglądów, część zaś Twoich wrażeń z przeczytanego tekstu.

[Następnie uczestnikom przydzielany był losowo tekst narracji kontrolnej lub eksperymentalnej]

**CZĘŚĆ ZASADNICZA**

Celem tego badania jest określenie, jakie poglądy i postawy mają czytelnicy Marka Krajewskiego. Zobaczysz kwestionariusz, który składa się z 40 stwierdzeń. Przeczytaj każde z nich uważnie i zaznacz przy poszczególnych stwierdzeniach, do jakiego stopnia zgadzasz się lub nie zgadzasz z każdym z nich.

Nie ma tu dobrych ani złych odpowiedzi, prosimy o szczere odpowiedzi.

PS.W pewnym miejscu umieściliśmy stwierdzenie kontrolne, w którym prosimy o zaznaczenie jednej, wskazanej przez nas odpowiedzi. Nie przeocz go, powodzenia!

**Skala odpowiedzi dla wszystkich stwierdzeń na stronach 1 – 4**

Zdecydowanie się nie zgadzam |1

Nie zgadzam się |2

Raczej się nie zgadzam |3

Ani się zgadzam, ani nie zgadzam |4

Raczej się zgadzam |5

Zgadzam się |6

Zdecydowanie się zgadzam |7

Strona 1 z 4

| 1 | Postrzegam siebie jako osobę lubiącą towarzystwo innych, aktywną i optymistyczną. |
| --- | --- |
| 2 | Kierunki humanistyczne dają równie dobre perspektywy, jak kierunki techniczne. |
| 3 | Zapłodnienie metodą in vitro jest niemoralne. |
| 4 | Postrzegam siebie jako osobę krytyczną względem innych, konfliktową. |
| 5 | Nasz kraj potrzebuje silnego przywódcy, po to by położyć kres radykalnym i niemoralnym prądom, które przeważają dziś w społeczeństwie. |
| 6 | Żywność genetycznie modyfikowana powinna być dopuszczona do obrotu handlowego. |
| 7 | Ubój delfinów i wielorybów powinien być natychmiast wstrzymany, nawet jeśli oznaczałoby to utratę pracy przez niektórych ludzi. |
| 8 | Nasz kraj potrzebuje wolnomyślicieli, którzy będą mieli odwagę przeciwstawić się tradycyjnym obyczajom, nawet jeśli nie spodoba się to wielu ludziom. |
| 9 | Postrzegam siebie jako osobę sumienną, zdyscyplinowaną. |
| 10 | „Staroświeckie obyczaje” i „staroświeckie wartości” wciąż pokazują najlepszy sposób życia. |

Strona 2 z 4

| 11 | Nasze społeczeństwo miałoby się lepiej, gdybyśmy okazywali tolerancję i zrozumienie dla nietradycyjnych wartości i opinii. |
| --- | --- |
| 12 | Polscy obywatele powinni mieć większy dostęp do broni niż obecnie. |
| 13 | Cierpienie zwierząt jest dopuszczalną ceną za wynajdywanie leków dla ludzi. |
| 14 | Postrzegam siebie jako osobę pełną niepokoju, łatwo wpadającą w przygnębienie. |
| 15 | Boskie prawa dotyczące aborcji, pornografii i małżeństwa muszą być ściśle przestrzegane, zanim będzie za późno, a ich naruszenia powinny być karane. |
| 16 | Powinno się wspierać i chronić mniejszości kulturowe. |
| 17 | Jestem za legalizacją miękkich narkotyków. |
| 18 | Potrzeby ludzkie zawsze powinny być ważniejsze od potrzeb zwierząt. |
| 19 | Postrzegam siebie jako osobę otwartą na nowe doznania, w złożony sposób postrzegającą świat. |
| 20 | Bardziej niż silnego przywódcy społeczeństwo potrzebuje otwartości wobec ludzi myślących inaczej – świat nie jest szczególnie zły lub niebezpieczny. |

Strona 3 z 4

| 21 | Byłoby najlepiej, gdyby gazety były cenzurowane w celu uniemożliwienia ludziom kontaktu ze szkodliwymi czy też odpychającymi treściami. |
| --- | --- |
| 22 | Wielu dobrych ludzi przeciwstawia się państwu, krytykuje Kościół i ignoruje „normalny sposób życia”. |
| 23 | Postrzegam siebie jako osobę zamkniętą w sobie, wycofaną i cichą. |
| 24 | Naszych przodków powinno się bardziej doceniać za to, jak zbudowali nasze społeczeństwo, z drugiej strony zaś powinniśmy położyć kres siłom, które to społeczeństwo niszczą. |
| 25 | Służba zdrowia powinna być sprywatyzowana. |
| 26 | Czuję się osobiście odpowiedzialny(a) za pomoc potrzebującym zwierzętom |
| 27 | Ludzie powinni przywiązywać mniej wagi do Biblii czy religii, a zamiast tego powinni wynajdywać swoje własne standardy moralne. |
| • | Pozycja kontrolna. Zaznacz odpowiedź „Raczej się zgadzam” |
| 28 | Postrzegam siebie jako osobę zgodną, życzliwą. |
| 29 | Jest wielu radykalnych, niemoralnych ludzi próbujących wszystko zrujnować – społeczeństwo powinno ich powstrzymać. |
| 30 | Chciałbym aby w Polsce wprowadzono EURO zamiast złotówki. |

Strona 4 z 4

| 31 | Niskie koszty produkcji pożywienia nie uzasadniają hodowania zwierząt w złych warunkach. |
| --- | --- |
| 32 | Postrzegam siebie jako osobę źle zorganizowaną, niedbałą. |
| 33 | Sądzę, że lepiej jest zaakceptować niemoralną literaturę, niż ją cenzurować. |
| 34 | Fakty pokazują, że po to by utrzymać prawo i porządek, powinniśmy być ostrzejsi wobec przestępczości oraz wobec niemoralności seksualnej. |
| 35 | Uważam, że małpom człekokształtnym powinno się przyznać prawa podobne do praw człowieka. |
| 36 | Postrzegam siebie jako osobę niemartwiącą się, stabilną emocjonalnie. |
| 37 | Sytuacja dzisiejszego społeczeństwa poprawiłaby się, gdyby ludzie sprawiający kłopoty byli traktowani z rozsądkiem i po ludzku. |
| 38 | Postrzegam siebie jako osobę trzymającą się schematów, biorącą rzeczy wprost. |
| 39 | Ludzie mają prawo posługiwać się zwierzętami wedle swego uznania. |
| 40 | Jeśli społeczeństwo tak chce, to obowiązkiem każdego prawdziwego obywatela jest pomóc wyplenić zło, które zatruwa nasz kraj od środka. |

**PYTANIA DO TEKSTU**

Teraz prosimy o odpowiedź na pytania dotyczące przeczytanego przez Ciebie na początku fragmentu powieści Marka Krajewskiego. Proszę zaznacz liczbę, która najlepiej odpowiada Twojej opinii na temat tego tekstu.

**Skala odpowiedzi dla wszystkich stwierdzeń**

Zdecydowanie się nie zgadzam |1

Nie zgadzam się |2

Raczej się nie zgadzam |3

Ani się zgadzam, ani nie zgadzam |4

Raczej się zgadzam |5

Zgadzam się |6

Zdecydowanie się zgadzam |7

| Wersja dla grupy eksperymentalnej | |
| --- | --- |
| 1 | Kiedy czytałem tekst, łatwo mi było wyobrazić sobie wydarzenia, które były w nim opisywane. |
| 2 | Kiedy czytałem tekst, zwracałem uwagę na to, co działo się w pomieszczeniu, w którym akurat się znajdowałem. |
| 3 | Mogłem wyobrazić sobie samego siebie w miejscu wydarzeń opisanych w tekście. |
| 4 | Byłem zaangażowany myślami w tekst, kiedy go czytałem. |
| 5 | Kiedy skończyłem czytać tekst, łatwo mi było przestać o nim myśleć. |
| 6 | Chciałem się dowiedzieć, jak tekst się skończy. |
| 7 | Tekst poruszył moje emocje. |
| 8 | Zastanawiałem się nad tym, jak inaczej mógłby się skończyć ten tekst. |
| 9 | Kiedy czytałem tekst, myślami byłem gdzie indziej. |
| 10 | Wydarzenia przedstawione w tekście są istotne z punktu widzenia mojego codziennego życia. |
| 11 | Wydarzenia przedstawione w tekście zmieniły moje życie. |
| 12 | Czytając tekst miałem przed oczyma wyraźny obraz małpki . |
| 13 | Czytając tekst miałem przed oczyma wyraźny obraz cyrku. |

| Wersja dla grupy kontrolnej | |
| --- | --- |
| 1 | Kiedy czytałem tekst, łatwo mi było wyobrazić sobie wydarzenia, które były w nim opisywane. |
| 2 | Kiedy czytałem tekst, zwracałem uwagę na to, co działo się w pomieszczeniu, w którym akurat się znajdowałem. |
| 3 | Mogłem wyobrazić sobie samego siebie w miejscu wydarzeń opisanych w tekście. |
| 4 | Byłem zaangażowany myślami w tekst, kiedy go czytałem. |
| 5 | Kiedy skończyłem czytać tekst, łatwo mi było przestać o nim myśleć. |
| 6 | Chciałem się dowiedzieć, jak tekst się skończy. |
| 7 | Tekst poruszył moje emocje. |
| 8 | Zastanawiałem się nad tym, jak inaczej mógłby się skończyć ten tekst. |
| 9 | Kiedy czytałem tekst, myślami byłem gdzie indziej. |
| 10 | Wydarzenia przedstawione w tekście są istotne z punktu widzenia mojego codziennego życia. |
| 11 | Wydarzenia przedstawione w tekście zmieniły moje życie. |
| 12 | Czytając tekst miałem przed oczyma wyraźny obraz dworcowej restauracji. |
| 13 | Czytając tekst miałem przed oczyma wyraźny obraz taksówkarza. |

**METRYCZKA**

Dziękujemy za wszystkie odpowiedzi. Pozostało jeszcze kilka pytań metryczkowych.

**Zaznacz wielkość miejscowości w której aktualnie (głównie) mieszkasz.**

1. Wieś
2. Miasto do 25 tys. mieszkańców
3. Miasto 25 - 50 tys. mieszkańców
4. Miasto 51 - 100 tys. mieszkańców
5. Miasto 101 - 200 tys. mieszkańców
6. Miasto 201 - 500 tys. mieszkańców
7. Miasto powyżej 500 tys. mieszkańców

**Jakie jest Twoje wykształcenie?**

1. Podstawowe/gimnazjum/ zawodowe
2. Średnie/ policealne
3. Wyższe/Licencjat

**Zaznacz TAK lub NIE na poniższe pytania. Czy…?**

|  | Odp. | Tak | Nie |
| --- | --- | --- | --- |
| 1 | Posiadasz rodzeństwo | 1 | 2 |
| 2 | Posiadasz samochód | 1 | 2 |
| 3 | Posiadasz zwierzęta domowe | 1 | 2 |
| 4 | Uprawiasz regularnie sport | 1 | 2 |
| 5 | Jesteś abstynentem | 1 | 2 |
| 6 | Jesteś wegetarianinem | 1 | 2 |
| 7 | Jesteś weganinem | 1 | 2 |
| 8 | Kryminały to mój NAJBARDZIEJ ulubiony gatunek literacki | 1 | 2 |
| 9 | Ogólnie rzecz biorąc, cieszę się dobrym zdrowiem | 1 | 2 |
| 10 | Czy kiedykolwiek doznałeś jakichś dotkliwych urazów fizycznych? | 1 | 2 |

**Jeśli masz rodzeństwo**

**Podaj proszę płeć i wiek rodzeństwa (jeśli masz kilkoro rodzeństwa, wymień po przecinku)**

......................

**Jeśli masz samochód**

**Podaj proszę markę samochodu i od jak długo go posiadasz**

......................

**Jeśli masz zwierzęta**

**Podaj proszę jakie zwierzęta posiadasz i od jakiego czasu**

......................

**Jeśli uprawiasz sport**

**Jaki sport uprawiasz i od jakiego czasu?**

......................

**Jeśli miałeś/aś wypadek**

**W wyniku czego doznałeś(aś) dotkliwych obrażeń fizycznych?**

1. wypadek lokomocyjny
2. działanie substancji chemicznych
3. działnie maszyn
4. atak zwierząt
5. inne, jakie? ..............

**KONKURS**

To już wszystkie pytania, dziękujemy za Twój wysiłek! Teraz przejdźmy do przyjemności :)

Aby wziąc udział w konkursie, prosimy Cię o wypełnienie poniższego formularza i odpowiedź na pytanie konkursowe.

Twój adres e-mail: ..............................

Imię i nazwisko: .........................

[] Oświadczam, że zapoznałem/am się i akceptuję Regulamin konkursu „Badanie profilu psychologicznego czytelników Marka Krajewskiego ” oraz wyrażam zgodę na przetwarzanie danych osobowych, zawartych w formularzu zgłoszeniowym, przez IMAS International Sp. z o.o. z siedzibą we Wrocławiu, ul. Braci Gierymskich 156, wyłącznie w celu i zakresie niezbędnym dla przeprowadzenia konkursu - nie dłużej niż przez okres przeprowadzania konkursu i do przedawnienia ewentualnych roszczeń.

Pytanie konkursowe brzmi:

Ile ofiar pochłonął tytułowy Władca Liczb? 5, 6, 8,9, 16, 17?

Dziękujemy za udział w ankiecie!
