# Supplementary material for: Literary Fiction Influences Attitudes Toward Animal Welfare
Source: PLoS One. 2016 Dec 22;11(12):e0168695. doi: 10.1371/journal.pone.0168695 (PMC5179074; doi:10.1371/journal.pone.0168695)
Supplement: S5 Text — Scale A in S5 Text provides an English translation of the original Polish scale used in the study. Scale B in S5 Text B provides the original Polish version. (DOCX) [file pone.0168695.s005.docx]

**S5 Text. ATAW Scale**

**Scale A: an English translation of the original Polish scale used in the study.**

1. The slaughter of whales and dolphins should be immediately stopped even if it means that some people will be put out of work
2. The suffering of animals is an acceptable price for inventing drugs for humans.*
3. Human needs should always come before the needs of animals*
4. I feel personally responsible for helping animals in need.
5. The low costs of food production do not justify maintaining animals under poor conditions.
6. Apes should be granted rights similar to human rights.
7. Basically, humans have the right to use animals as we see fit.*

**Scale B: the original Polish version of the scale used in the study**

1. Ubój delfinów i wielorybów powinien być natychmiast wstrzymany, nawet jeśli oznaczałoby to utratę pracy przez niektórych ludzi.
2. Cierpienie zwierząt jest dopuszczalną ceną za wynajdywanie leków dla ludzi.*
3. Potrzeby ludzkie zawsze powinny być ważniejsze od potrzeb zwierząt.*
4. Czuję się osobiście odpowiedzialny(a) za pomoc potrzebującym zwierzętom
5. Niskie koszty produkcji pożywienia nie uzasadniają hodowania zwierząt w złych warunkach.
6. Uważam, że małpom człekokształtnym powinno się przyznać prawa podobne do praw człowieka.
7. Ludzie mają prawo posługiwać się zwierzętami wedle swego uznania.*
